# Multi-characterization of LiCoO_2_ cathode films using advanced AFM-based techniques with high resolution

**DOI:** 10.1038/s41598-017-11623-0

**Published:** 2017-09-18

**Authors:** Jiaxiong Wu, Shan Yang, Wei Cai, Zhuanfang Bi, Guangyi Shang, Junen Yao

**Affiliations:** 1 Department of Applied Physics, Beihang University, Beijing, 100191 People’s Republic of China; 2 Key Laboratory of Micro-nano Measurement-Manipulation and Physics (Ministry of Education), Beihang University, Beijing, 100191 People’s Republic of China; 3 Department of Mechanical Engineering, National University of Singapore, Singapore, 117576 Singapore

## Abstract

The thin film Li-ion batteries have been extensively used in micro-electronic devices due to their miniaturization, high capacity density and environmental friendliness, etc. In order to further prolong the lifetime of the film batteries, one of important tasks is to explore the aging mechanisms of the cathode films. In this paper, we especially focused on the multi-characterization of the LiCoO_2_ film in nanoscale, which is carried out by combining advanced AFM-based techniques with capacity measurement. The surface morphology, contact stiffness as well as surface potential were measured by amplitude modulation-frequency modulation (AM-FM) and kelvin probe force microscope (KPFM), respectively. Remarkable changes after different numbers of charge/discharge cycling were observed and the intrinsic reasons of them were discussed in detail. To acknowledge the relationship with these microscopic changes, the macro-capacity of the thin films was also measured by the galvanostatic charge/discharge method. These comprehensive results would provide a deep insight into the fading mechanism of the cathode film, being helpful for the design and selection of the cathode film materials for high performance batteries.

## Introduction

Lithium ion batteries are important energy storage devices and have been widely applied in lots of fields, such as the electric grid, cellphone and electric vehicles^1–3^. Among various kinds of Li-ion batteries, due to miniaturization, high capacity density and environmental friendliness, etc., the thin film Li-ion batteries can be used in the micro-electronic devices, such as chips in computer, micro-medical devices and intelligent labels^4,5^. With increasing demand for the thin film batteries, improving the performance of them have attracted more and more interests recently. Besides developing new electrode materials, better understanding the aging mechanism of the thin film batteries is an important issue.

Numerous studies indicated that the thin film batteries aging depends on the changes in structure and properties of the electrode film, which had been successfully characterized by lots of techniques, such as scanning electron microscope (SEM)^6,7^, transmission electron microscope (TEM)^8,9^, X-ray diffraction (XRD)^10–13^, X-ray photoelectron spectroscopy (XPS)^14–16^, electrochemical impedance spectroscopy (EIS)^17,18^ and so on. However, these characterization techniques are unidirectional in the meaning that only one or a small part of the whole properties of the electrode material can be probed. Thanks to the rapid development of AFM-based techniques^19–21^, the surface structure and properties of the battery electrode can be investigated simultaneously and with higher spatial resolution.

Mechanical properties are crucial for maintaining structural integrity and morphology stability of the electrodes. Several techniques, including nano-indentation, contact resonance atomic force microscope (CRAFM) and amplitude modulation-frequency modulation (AM-FM), had been used to study the mechanical properties of the film electrode, such as stiffness and elastic modulus. The nano-indentation is the most widely used method, by which the elastic modulus, hardness, stiffness of electrode material, like LiMn_2_O_4_, RuO_2_ and TiO_2_, had been measured^22–24^. Unfortunately, the nano-indentation method would destruct the sample surface and provide the information only on micro-scale. By comparison, CRAFM is a non-destructive method with a higher spatial resolution, i.e. in nanoscale, and had also been applied in measurements of the mechanical properties in kinds of materials^25–27^. However, during the CRAFM scanning, the tip is in continuous contact with the surface, leading to wear of the tip and change of the resonance frequency, consequently affecting the accuracy of the results^28^. Amplitude modulation and frequency modulation (AM-FM), firstly proposed by Garcia Ricardo and Proksch Roger^29^, could prevent from the wear of the tip due to working in tapping mode. Moreover, this technique has much higher resolution than that of the nano-indentation technique. The most important thing is, surface morphology and information related to surface mechanical properties can be obtained at the same time by this method^30^, which had been successfully applied in the characterization of different kinds of materials, such as polymers, biomaterials and electrode materials^29–31^. Especially, Yang *et al*. had studied changes in stiffness of the Li-rich cathode thin film by the AM-FM technique^32^, in which the stiffness after charging and discharging were measured separately in the first cycle. However, those results only displayed the information on the stiffness changes in the first cycling. In order to better understand the aging mechanism of the cathode, an important issue to be addressed is to study the stiffness after repeated cycles.

Surface electrical properties are also important for the electrode material because they are closely related to the electron transport and Li-ion diffusion. The conductive atomic force microscope (CAFM) was usually applied to measure the surface electronic conductivity of the electrode material^20,33,34^. While, the kelvin probe force microscope (KPFM) is capable of obtaining the surface potential, which contains more information besides of the electronic conductivity^35^, and has been successfully applied to measure variations of the surface potential at micro- and nano-scale in various materials, such as semiconductor^36,37^, organic material^38,39^, battery electrode^35,40,41^, and so on. By KPFM, Nagpure *et al*. studied the surface potential of LiFePO_4_ electrode only in the situations of aged and unaged^42^, rather than that after different numbers of charge/discharge cycling. Since the aging mechanism of the thin film Li-ion batteries is complicated, and both the surface morphology and the corresponding properties depend on the electrochemical history, the intermediate processes of charging/discharging are worthy to be traced.

In this work, AM-FM, KPFM and galvanostatic charge/discharge methods were combined to study the effect of charge/discharge cycles on variations of the surface structure and properties of the cathode film for deeply understanding the aging mechanism of the thin film Li-ion batteries. We particularly prepared the LiCoO_2_ thin film (see Fig. S1 in supplementary information) as the battery cathode, because it not only is a widely used cathode material with high theoretical capacity (272 mAh/g) but also is free of conducting additive and binder, excluding any possible influence of carbon and binder^43^. The surface morphology and contact stiffness of the cathode film were investigated simultaneously by the AM-FM technique. Meanwhile, the surface morphology and surface potential of the cathode film were measured by KPFM. In order to obtain more information during the aging processes in detail, variations of the surface morphology, stiffness and surface potential were characterized before cycling and after 10, 50, 100 charge/discharge cycles, respectively. The capacity fading of the film was also studied by the galvanostatic charge/discharge method. These experimental results, obtained by the combination of the AFM-based techniques and macro-capacity measurement show what happened at different cycles and establish the relationship between the aging mechanism and variations of the surface structure and properties of the cathode films.

## Results

### The AM-FM and KPFM techniques

**Figure 1:**
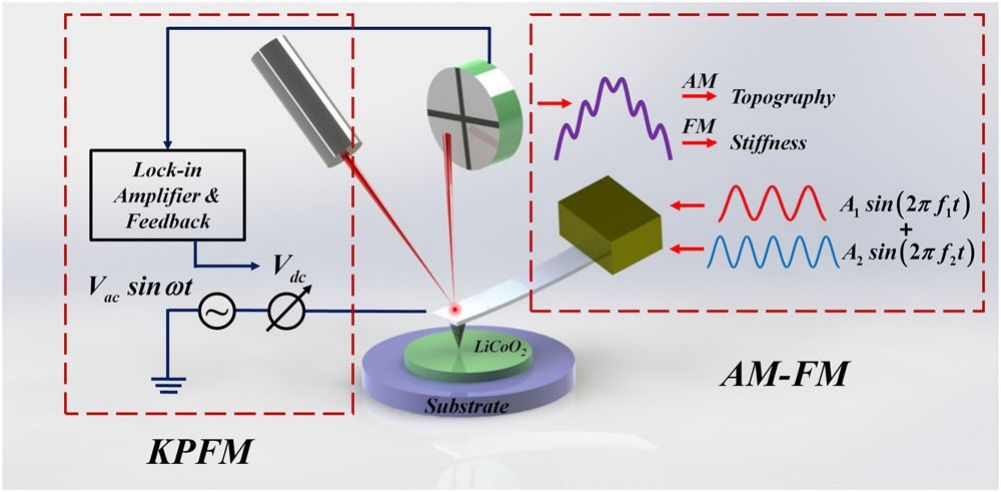
The schematic of AM-FM and KPFM techniques.

The schematic of the AM-FM and KPFM techniques employed in this study is shown in Fig. 1. For AM-FM operation, the cantilever is excited simultaneously at two resonance frequencies: the fundamental *f*_1_ and a higher order *f*_*n*_, where *n* is the mode number, and the second order resonance frequency *f*_2_ is generally adopted. Same as the standard tapping mode, *f*_1_ is used for topographic imaging by means of amplitude modulation. And since *f*_2_ is sensitive to the elastic tip-sample interaction^29^, it is applied to obtain the information of the surface elasticity by the method of frequency modulation. In the AM-FM measurement, the tip-sample contact stiffness *k*_*ts*_ is related to the second resonance frequency shift *Δf*_2_ and given by the following equation (1)^29,32^

1 $${k}_{ts}\approx \frac{2{k}_{2}{\rm{\Delta }}{f}_{2}}{{f}_{0,2}}$$

where *k*_2_ is the spring constant of cantilever at *f*_2_, and *f*_*0*,2_ is the second resonant frequency measured at a free or reference position. By measuring the frequency shift *Δf*_*2*_ and calibrating the parameters of the cantilever, the tip-sample stiffness *k*_*ts*_ can be obtained. In the experiment, *f*_1_ is excited with the voltage of 2 V by the MFP-3D controller (Asylum Research, CA, USA) and *f*_2_ is driven with the voltage of 10–20 mV by the PLL, respectively. It should be noticed that the amplitude of the second resonance *f*_2_ is much smaller than that of the fundamental resonance *f*_1_, in order to prevent perturbation to the fundamental cantilever dynamics.

It is known that the electrostatic contact potential difference is generated due to difference of the work function between a conductive tip and a sample surface. The surface contact potential difference (*V*_*CPD*_) is written as the equation (2)^35,42^.

2 $${V}_{CPD}=\frac{{E}_{tip}-{E}_{sample}}{e}$$

where *E*_*tip*_ and *E*_*sample*_ represent the work function of the tip and the sample respectively, and *e* is the charge of one electron. When KPFM operates, an oscillating voltage (*V*_*ac*_ *sinωt*) is applied to the tip during scanning and the electrostatic force between the tip and the sample is detected by the lock-in amplifier. If an external voltage (*V*_*dc*_) is adjusted to nullify the electrostatic force by the potential feedback controller, it exactly equals to the potential of the sample surface. During measurement, KPFM operates in two trace scanning. In the first scanning, the surface topography is obtained as same as the standard AFM tapping mode. In the second trace, the conductive tip is lifted up to a fixed distance from the surface to measure the surface potential difference. Hence, the AM-FM and KPFM techniques are capable to implement a multi-characterization, including surface morphology, contact stiffness and surface potential of the LiCoO_2_ cathode film in nanoscale.

### Surface morphology and contact stiffness evolutions

**Figure 2:**
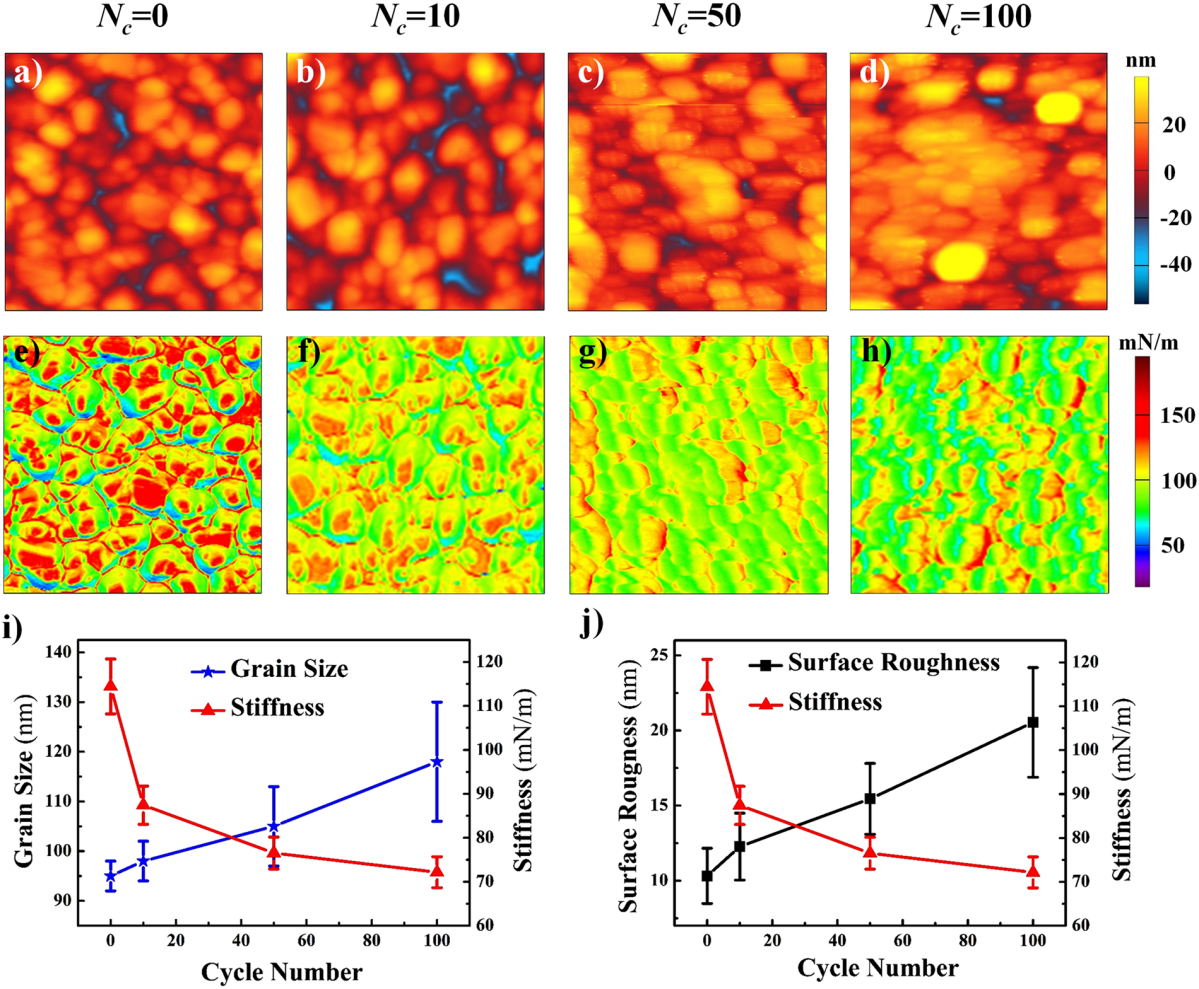
The LiCoO_2_ cathode thin film images and statistic data by AM-FM: (**a–d**) surface morphology; (**e–h**) contact stiffness after different cycles, *N*_*c*_ stands for the charge/discharge cycle number, the scan size is 1 μm × 1 μm. (**i**) Grain size and stiffness; (**j**) RMS surface roughness and stiffness with different cycle number.

The surface morphology and contact stiffness of the LiCoO_2_ cathode film were directly investigated by using the AM-FM technique simultaneously. Such experiments were performed before and after charge/discharge cycling, respectively. The typical topographic images of the sample are given in Fig. 2(a–d), showing a surface morphologic evolution with different cycle numbers. This change was quantitatively calculated in terms of the average grain size and the surface roughness (root mean square, RMS), analyzed from the imaging area. As shown in Fig. 2(i,j), the average grain sizes are 95.6 ± 3.9 nm, 98.6 ± 4.2 nm, 102.7 ± 7.6 nm and 115.2 ± 13.4 nm obtained by imaging software (Image J), as well as the RMS surface roughness values are 10.3 ± 1.8 nm, 12.3 ± 2.2 nm, 15.5 ± 2.4 nm and 20.5 ± 3.7 nm after 0, 10, 50 and 100 cycles, respectively. These results indicate that both the grain size and the surface roughness increase with the increase of cycle number. These phenomena are well in agreement with that of LiMn_2_O_4_, which was reported previously^24^.

The contact stiffness maps before and after charge/discharge cycling are given in Fig. 2(e–h), where a significant change in the stiffness can be clearly seen and quantitatively characterized by RMS. As given in Fig. 2(i) and (j), the stiffness of the film is 114.4 mN/m before cycling. The contact stiffness then decreases by 23.6%, 12.5% and 5.7% after 10, 50 and 100 cycles, respectively. Obviously, the rate of the change is relative large after 10 cycles, while getting small after 50 cycles and smaller after 100 cycles. It should be noted that there are much difference in the stiffness between grain boundaries and grain interior, which may be resulted from the different Li-ion concentrations^31^.

### Surface morphology and potential distribution evolutions

**Figure 3:**
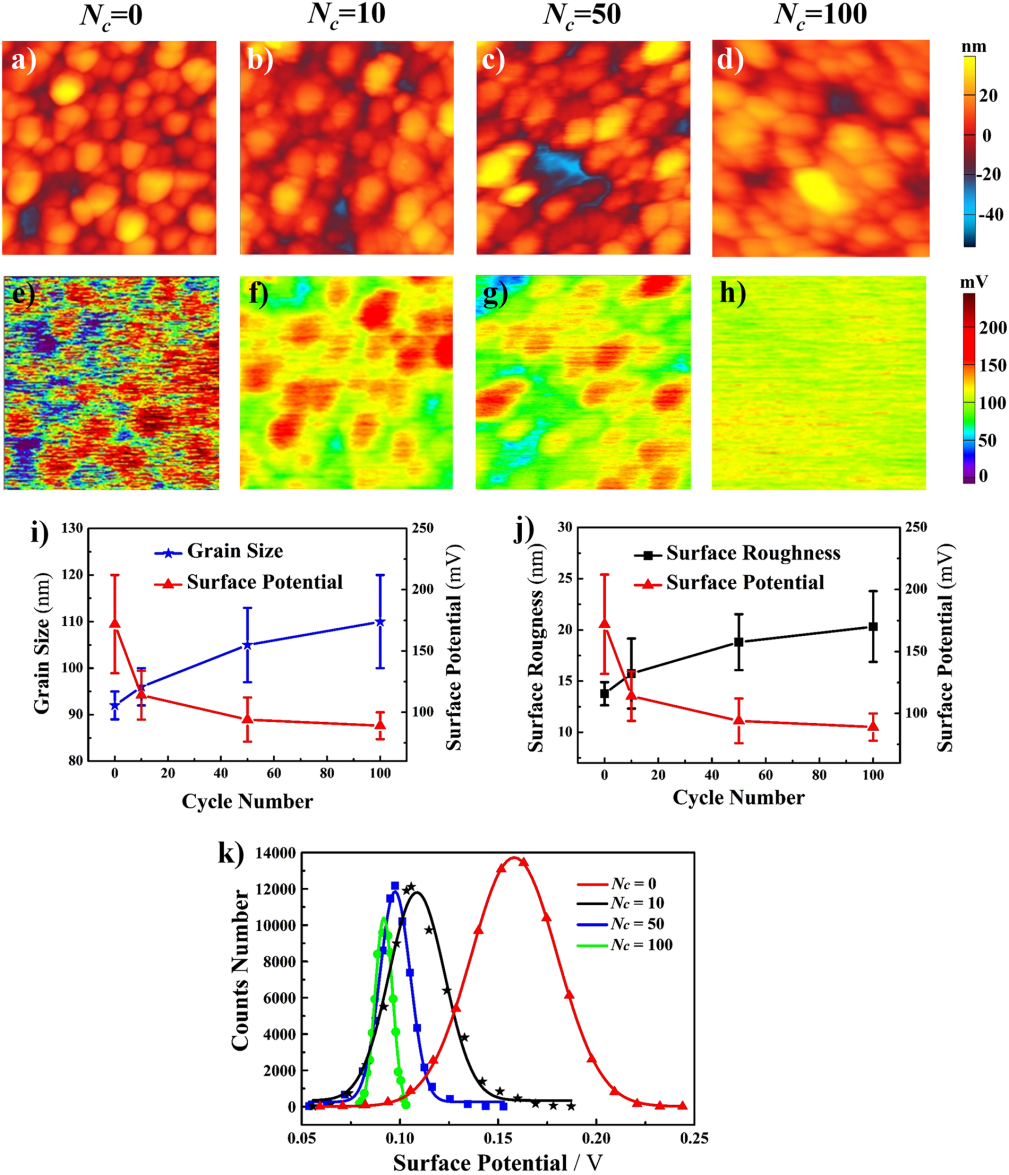
The LiCoO_2_ cathode thin film images and statistic data by KPFM: (**a–d**) surface morphology; (**e–h**) surface potential after different cycles, *N*_*c*_ stands for the charge/discharge cycle number, the scan size is 1 μm × 1 μm. (**i**) Grain size and surface potential; (**j**) RMS surface roughness and surface potential; (**k**) surface potential distribution with different cycle number.

Figure 3(a–h) show the surface topographic and potential maps of the sample before and after cycling, respectively. The same tip was used in the entire process in order to keep the work function of the tip being constant. From Fig. 3(a–d), the surface morphologic changes can be observed and their quantitatively analyzed data are given in Fig. 3(i,j), showing increment of both the grain size and surface roughness. These results are well in agreement with those given in Fig. 2(i,j). Figure 3(e–h) show that remarkable changes exist in the surface potential of the sample during the charge/discharge process. It can be easily seen that the surface potential of the film before cycling is non-uniformly distributed, while it is getting more and more uniform with the increase of cycle number. As the quantitative results given in Fig. 3(i,j), the RMS surface potential values are 172 mV before cycling and 114 mV, 94 mV and 89 mV after 10, 50 and 100 cycles, that is to say, decreased by 33.7%, 17.5% and 5.3% after 10, 50 and 100 cycles, respectively. These data indicate that the decrement in the surface potential after10 cycles is more significant than that for 50 and 100 cycles.

The surface potential images discussed above are generated by a matrix of 256 × 256 data points, and the distribution of these data points are shown in Fig. 3(k). For each cycle, a histogram is created with 17 equally spaced bins based on the Sturges’s rule^44^. To obtain the distribution curves, a normal probability density distribution function was adopted for data fitting, which is given in the following equation (3).

3 $$f(x)=\frac{1}{\sqrt{2\pi {\sigma }^{2}}}{e}^{-\frac{{(x-\mu )}^{2}}{2{\sigma }^{2}}}$$

where *μ* and *σ* are the mean and the standard deviation of the data points, respectively. In Fig. 3(k), it is clearly shown that the surface potential distribution of the pristine film is non-uniform. According to the data fitting results, the standard deviation are 16.45 mV, 10.65 mV, 5.8 mV and 3.4 mV after 0, 10, 50 and 100 cycles, respectively, which indicate that the surface potential distribution become almost completely uniform after 100 cycles.

### Galvanostatic charge/discharge measurenents

**Figure 4:**
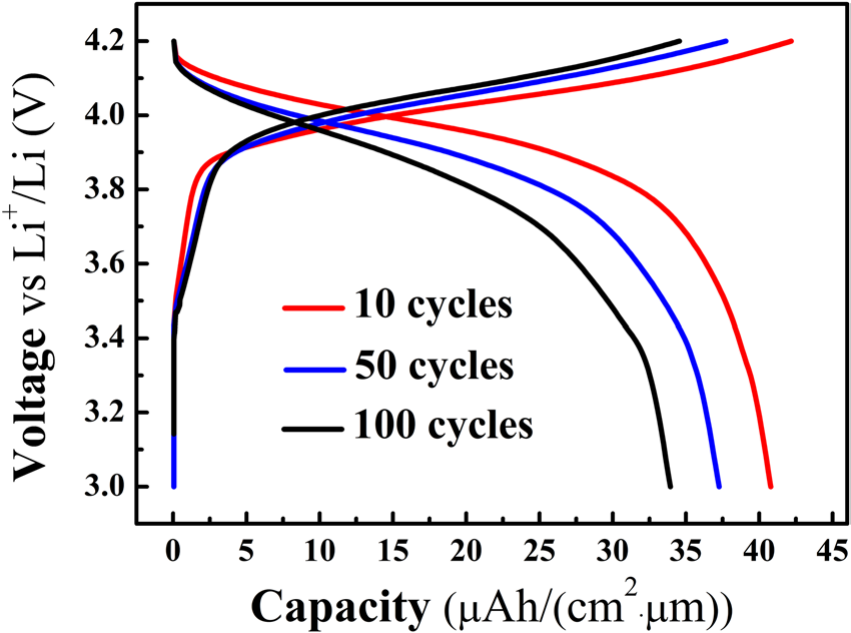
Charge/discharge capacity curves of the LiCoO_2_ film measured by the galvanostatic charge/discharge method.

In order to bulid up a relationship between the macro- and the micro-scopic evolutions, the capacity of the LiCoO_2_ cathode film was also measured by the galvanostatic charge/discharge method after 10, 50 and 100 cycles, respectively. The measurements were performed in the range of 3.0–4.2 V at the constant current density of 10 μA/cm^2^. The results are given in Fig. 4, where each curve shows a typical charge/discharge plateau roughly ranged from 3.9 V to 4.1 V. This range corresponds to the Li^+^ intercalation/de-intercalation processes during reversible electrochemical reactions of the LiCoO_2_ cathode film^45^. In Fig. 4, the discharge capacity of the film after 10, 50 and 100 cycles are ~41 μAh/(cm^2^·μm), 37 μAh/(cm^2^·μm) and 34 μAh/(cm^2^·μm), respectively. Since the density of the LiCoO_2_ material is 5.1 g/cm^3^, the corresponding discharge capacity of the film should be 80.4 mAh/g, 72.5 mAh/g and 66.7 mAh/g, respectively. Furthermore, the initial discharge capacity is ~48 μAh/(cm^2^·μm) and 94.1 mAh/g. The rate of the decrement of the discharge capacity after 10, 50 and 100 cycles are 14.5%, 9.8% and 8%, respectively. It is easily deduced that the decrease in capacity is more significant after 10 cycles than that after 50 and 100 cycles.

## Discussion

The topographic images in Figs 2(a–d) and 3(a–d) obtained by AM-FM and PKFM respectively, demonstrate systematic morphologic change of the LiCoO_2_ cathode film with different charge/discharge cycle numbers. Quantitatively, average grain sizes in the film almost smoothly increased from 95.6 nm to 115.2 nm with the increase of cycle number, as seen in Figs 2(i) and 3(i). These changes of grain size are induced by the change of lattice structure of LiCoO_2_ due to Li^+^ intercalation/de-intercalation during charge/discharge processes. It is reported that the lattice parameter of LiCoO_2_ expanded up by 1.9% along *c* axis, while slightly contracted along *a* axis during charge/discharge processes^46,47^. Based on this anisotropic change of lattice structure, the grains are physically bound by the neighbors, inducing the stress among the grains. In addition, due to redistribution of Li^+^ in the cathode film during cycling, various sizes of the grains have different volume changes, which also contribute to the generation of stress between grains. Furthermore, when Li^+^ content in the cathode film is excessively extracted locally, defects are generally formed in the LiCoO_2_ grains, resulting in the formation of stress as well^48^. The total stress in the film should be the superposition of the three stress discussed above, and will accumulate with the increase of cycle numbers, which may cause the fracture of the grains. These fractured smaller nanograins are agglomerated, leading to the continues increase of grain size. Hence, the enlargement of grain size and the increment of grain coarseness can be explained by the fragmentation of grains^49^ and the agglomeration of fractured smaller nanograins due to the stress^24^. This explanation is confirmed by the statistical results of surface roughness in Figs 2(i,j) and 3(i,j), in which the grain size and RMS surface roughness increase with the increase of cycle number.

Figure 2(e–h) show remarkable change in the contact stiffness of the LiCoO_2_ film after charge/discharge cycling. In contrast to increase of the average grain sizes and the RMS surface roughness, the contact stiffness decrease with the increase of cycle number, as shown in Fig. 2(i,j). Since the stiffness of the film is in principle related to microstructure, elastic modulus of materials, film stress, etc., several factors responsible for the stiffness degradation of the LiCoO_2_ film should be considered. Firstly, the stiffness degradation is closely associated with the lithium content reduction in the film. It was reported that the contact stiffness of the Li_1.2_Co_0.13_Ni_0.13_Mn_0.54_O_2_ cathode film had a significant decreases after charging, while that of the film increased after discharging^32^. In the former process, the lithium content reduced, accompanied with the phase changes from the layered structure to spinel one, and in the latter process, the Li^+^ inserted back into the film. Due to the production of Li^+^ ionic resistance, the reduction of Li^+^ content in charging process is larger than the increase of that in discharging process^50^. These results imply that the reduction of lithium content is irreversible in such kind of layered cathode films. Thus, the irreversible reduction of lithium concentration is believed to be one important factor in the stiffness degradation of the LiCoO_2_ film after cycling. Secondly, change of the elastic modulus of materials will also affect the stiffness of them. When the cathode film is charged, Li^+^ is extracted from the film and the phase of LiCoO_2_ transforms from initial hexagonal phase (H1, LiCoO_2_) to a new hexagonal one (H2, Li_x_CoO_2_, 0.5 ≤ x < 1). And after the film is discharged, Li^+^ is inserted back into the film and the phase transforms to H1 again^51,52^. Since the elastic modulus of Li_x_CoO_2_ is lower than that of LiCoO_2_^53^, degradation of the contact stiffness occurs after charge/discharge cycling. Thirdly, transformation of the layer structure LiCoO_2_ (H1) to Li_x_CoO_2_ (H2) is always accompanied with Li^+^ extraction, inducing volume expansion of the film. Nevertheless, the volume expansion of the LiCoO_2_ film is constrained by the rigid Si substrate, leading to compressive stress in the film as well as stiffness degradation^24^. It should be noticed that the stiffness degradation is more significant after 10 cycles than that after 50 and 100 cycles. The phenomenon is expected to be related to the surface chemical reactions. It was reported that a solid electrolyte interphase (SEI) layer was formed after the first few cycles. The SEI layer is known to play an important role in elastic modulus degradation in the cathode film^54^. Hence, the SEI layer formation is a possible reason for the significant degradation of the film stiffness after 10 cycles.

The images in Fig. 3(e–h) and the curves in Fig. 3(i,j) show fairly distinct changes in the surface potential distribution after cycling. Same as the results of contact stiffness, the surface potential is significantly decreased with the increase of cycle number. Several reasons for the decrease should be considered as follows. The first one is related to the phase transition between LiCoO_2_ and Li_x_CoO_2_ during the charge/discharge processes. After cycling, these two phases coexist in the cathode film due to the irreversibility of Li^+^ intercalation and deintercalation. Since work function of LiCoO_2_ is higher than that of Li_x_CoO_2_^55^, the final surface potential decreased. The second one is associated with the surface electrochemical reactions, such as decomposition of the electrolyte and adsorption of some impurities on the film surface, which could alter the surface electrical properties^35,56^. The third one is coarsening of the nanograins, which was known to lead to an increase in the surface electronic resistance and a decrease in the surface conductivity, accordingly leading to the decrease of the surface potential^42^. In addition, it is worthy to notice that the surface potential is significantly decreased after 10 cycles. Similar results were also found when electronic conductivity of the LiCoO_2_ material was measured^57^, concluding that the surface potential distribution in the film tends to be uniform with the increase of the cycle numbers. Especially, more attention should be paid to the image in Fig. 3(h), which almost shows a completely uniform distribution in the surface potential after 100 cycles. These phenomena are possibly due to the redistribution of Li^+^ in the cathode film during the charge/discharge process, and the distribution of Li^+^ is getting more and more uniform with the increase of the cycle number^58^. The distribution of Li^+^ always effect that of electrons in the same film, and consequently the surface potential is uniformly distributed after 100 cycles.

It should be emphasized that, based on the results and discussion above, the relationship between macro-capacity and microscopic changes of the LiCoO_2_ cathode film can be built up by the combination of the galvanostatic measurement and AFM-based techniques. The relationship would provide direct insight into microscopic aging mechanism of the cathode material. It was shown that the aging mechanism of the LiCoO_2_ film are closely related to the nanograins coarsening, stiffness degradation and surface potential decrease, and all of them are simultaneous and irreversible. Moreover, these changes after the first few cycles are more significant than those after the subsequent cycling, which is coincidental with the decrease in the discharge capacity measured using the galvanostatic method. These results suggest that one important issue to be addressed is how to reduce the changes, especially the changes after the first few cycles. For example, how to reduce the stiffness degradation, because the cathode film with higher stiffness means a more stable structure, which is in favor of Li^+^ intercalation and de-intercalation.

## Conclusions

In summary, the surface morphology, contact stiffness and surface potential distribution of the LiCoO_2_ thin film were investigated by the AM-FM and KPFM methods, respectively. From the results, it was found that the grain size enlargement, stiffness degradation and surface potential reduction of the cathode film were simultaneous and irreversible with the increase of the cycle number. It is worthy to note that all of them changed more obviously after the first 10 cycles than after 50 and 100 cycles. The capacity of the LiCoO_2_ film was also measured by the galvanostatic charge/discharge method. By comparing the results of the contact stiffness, surface potential and capacity, we found that the capacity fading of cathode film is attributed to the degradation of the contact stiffness and the surface potential. Based on the results and discussion, the combination of the galvanostatic measurement and AFM-based techniques would provide a multi-characterization method, paving a way to investigate the aging mechanism of similar electrode materials for the development of the film batteries. In the following work, we will study the changes of the structure and the properties of the film, especially during the first 10 cycles, for getting more information of the aging mechanism in detail. Furthermore, in order to exclude the influence of the neighboring particles in the film, the morphology, contact stiffness and surface potential, etc. of a single particle will be studied.

## Method

### AFM-based setup

All of the characterizations were conducted by the commercial scanning probe microscopy (MFP-3D, Asylum Research, USA). Both the surface morphology and stiffness images were carried out by AM-FM experiments in tapping mode using a tip (AC240TM, Olympus, Japan) with a force constant of 2 N·m^−1^ and a resonance frequency of 70 kHz. Especially, the second eigenmode resonance frequency of the tip is 399.95 kHz. Before all characterization, the stiffness and inverse optical lever sensitivity of the cantilever were calibrated using Sader and thermal noise methods.

KPFM experiments were performed in tapping mode using a conductive probe (AC240TM, Olympus, Japan) with a force constant of 2 N·m^−1^ and a resonance frequency of 70 kHz. During the KPFM scanning process, an AC voltage *V*_*AC*_ = 3 V was applied to the tip and the tip was lifted up 40 nm from the sample surface. Furthermore, in order to eliminate the affection of contaminants on surface potential, the sample was rinsed by acetone and deionized water three times before KPFM characterization. Other scanning parameters were also optimized for high quality images. All KPFM measurements were conducted under ambient environment.

### Synthesis of LiCoO_2_ thin film electrode

Lithium acetate (CH_3_COOLi·H_2_O) and cobalt acetate ((CH_3_COO)_2_Co·4H_2_O) were dissolved in distilled water as Li and Co source in the molar ratio 1:1. Citric acid acted as chelating agent was dissolved in i-C_3_H_7_OH which was used to increase the wettability of the precursor solution. After that, the two solutions were mixed. PVP (polyvinylpyrrolidone) acted as thicker was added into the mixed solution under stirring. The molar ratio of composition for the LiCoO_2_ precursor was CH_3_COOLi:(CH_3_COO)_2_Co:PVP: Citric acid:i-C_3_H_7_OH:H_2_O = 1:1: 1:1:20:40.

LiCoO_2_ thin films were prepared via sol-gel spin coating on silicon substrate. Before coating, Au was sputtered on a silicon substrate to increase the conductivity of the surface. A drop of precursor solution was spin coated on the Au/Si substrate at 3000 rpm for 30 s. After that, the coated film was heated at 370 °C for 20 min. Subsequently, the formed thin film was crystallized at 750 °C for 8 h.

### Structural characterization

The crystal structure of the prepared LiCoO_2_ thin films were characterized by X-ray diffraction with Ni filtered CuKα (40 kV and 30 mA) at a scanning rate of 5° min^-1^ in the 2θ range from 10°–60° (Shimadzu, XRD-6000). The morphology of the thin film surface was characterized by the field-emission scanning electron microscope (Hitachi S-4800), which operated at the accelerating voltage of 20 kV. The structure of the film was also characterized by the high resolution transmission electron microscope(Tecnai G^2^ F30) with an acceleration voltage of 200 kV.

### Electrochemical Measurements

The thin film battery was assembled in argon-filled glove box with H_2_O and O_2_ concentration less than 0.1ppm. Self-made LiCoO_2_ thin film was used as the working electrode, and a piece of Li foil was used as the counter electrode and reference electrode. LiCoO_2_ thin film and Li foil were separated by two pieces of separators (Celgard 2500). The electrolyte was 1 M LiPF_6_ in 1:1 ethylene carbonate EC and diethyl carbonate (DEC) solution. Half battery assembling was conducted by Swaglok test cell. Charge/discharge cycling was carried out by using battery testing station (LAND-CT2001A), at the current density of 10 μA/cm^2^ with the voltage range between 3.0 V and 4.2 V at room temperature. In order to investigate the changes of the properties during charge/discharge cycling, the discharge capacity of the battery were measured in different cycle numbers and then the battery was dissembled. The dissembled LiCoO_2_ thin films were rinsed by acetone and ethanol three times for subsequent characterization.

## Electronic supplementary material

Supplementary Information
