# Supplementary material for: Multi-characterization of LiCoO2 cathode films using advanced AFM-based techniques with high resolution
Source: Sci Rep. 2017 Sep 18;7:11164. doi: 10.1038/s41598-017-11623-0 (PMC5603513; doi:10.1038/s41598-017-11623-0)
Supplement: Supplementary file 1 — Supplementary Information [file 41598_2017_11623_MOESM1_ESM.pdf]

Supplementary Information

Multi-characterization of $LiCoO_2$ cathode films using advanced AFM-based techniques with high resolution

Jiaxiong Wu[1,2], Shan Yang[3], Wei Cai[1,2], Zhuanfang Bi[1,2*], Guangyi Shang[1,2*] and Junen Yao[1,2]

[1]Department of Applied Physics, Beihang University, Beijing 100191, People's Republic of China.
[2]Key Laboratory of Micro-nano Measurement-Manipulation and Physics (Ministry of Education), Beihang University, Beijing 100191, People's Republic of China
[3]Department of Mechanical Engineering, National University of Singapore, 117576, Singapore
*Corresponding author email: bizhuanfang@buaa.edu.cn; gyshang@buaa.edu.cn

S1. Crystallinity and morphological measurements

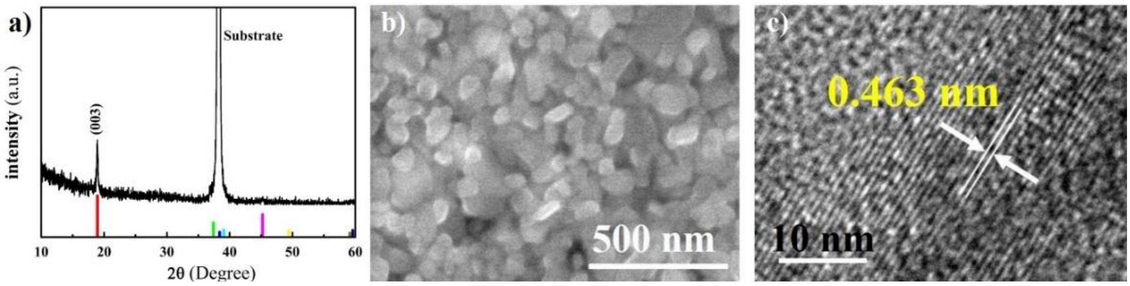

Figure S1. (a) XRD, (b) SEM and (c) HR-TEM characterization of the $LiCoO_2$ film.

In Fig. S1(a), besides the strong peak from the Au substrate, the (003) peak of the $LiCoO_2$ at 18.93° is clearly observed, which indicates a strongly preferred (003) orientation. Other peaks indexed as (101), (006), (012), (104) at 37.4°, 38.4°, 39.1°, 45.2° are overlaid by the substrate and cannot be distinguished. The results indicate that the film has a crystalline $LiCoO_2$ phase and layered structure. The surface morphology of the $LiCoO_2$ thin film characterized by SEM in Fig. S1(b) shows that the film consists of compacted nano-grains with homogeneous grain size of ~100 nm, which is well in agreement with the results in Fig. 2 and Fig. 3. To further verify the composition of the $LiCoO_2$ thin film, HR-TEM was applied to analyze the film and the result is shown in Fig. S1(c). Quantitatively analyzed results indicate that the distance of diffraction fringes is 0.463 nm, which is well indexed with the diffraction plane (003) in $LiCoO_2$.
